# Supplementary material for: The interactome of intact mitochondria by cross-linking mass spectrometry provides evidence for coexisting respiratory supercomplexes
Source: Mol Cell Proteomics. 2017 Dec 8;17(2):216–32. doi: 10.1074/mcp.RA117.000470 (PMC5795388; doi:10.1074/mcp.RA117.000470)
Supplement: Supplemental Data [file supp_RA117.000470_133922_0_supp_23978_fzffwf.zip › spectra_annotation/mito_spectra_annotation/222-2-2-1-2-1.pdf]

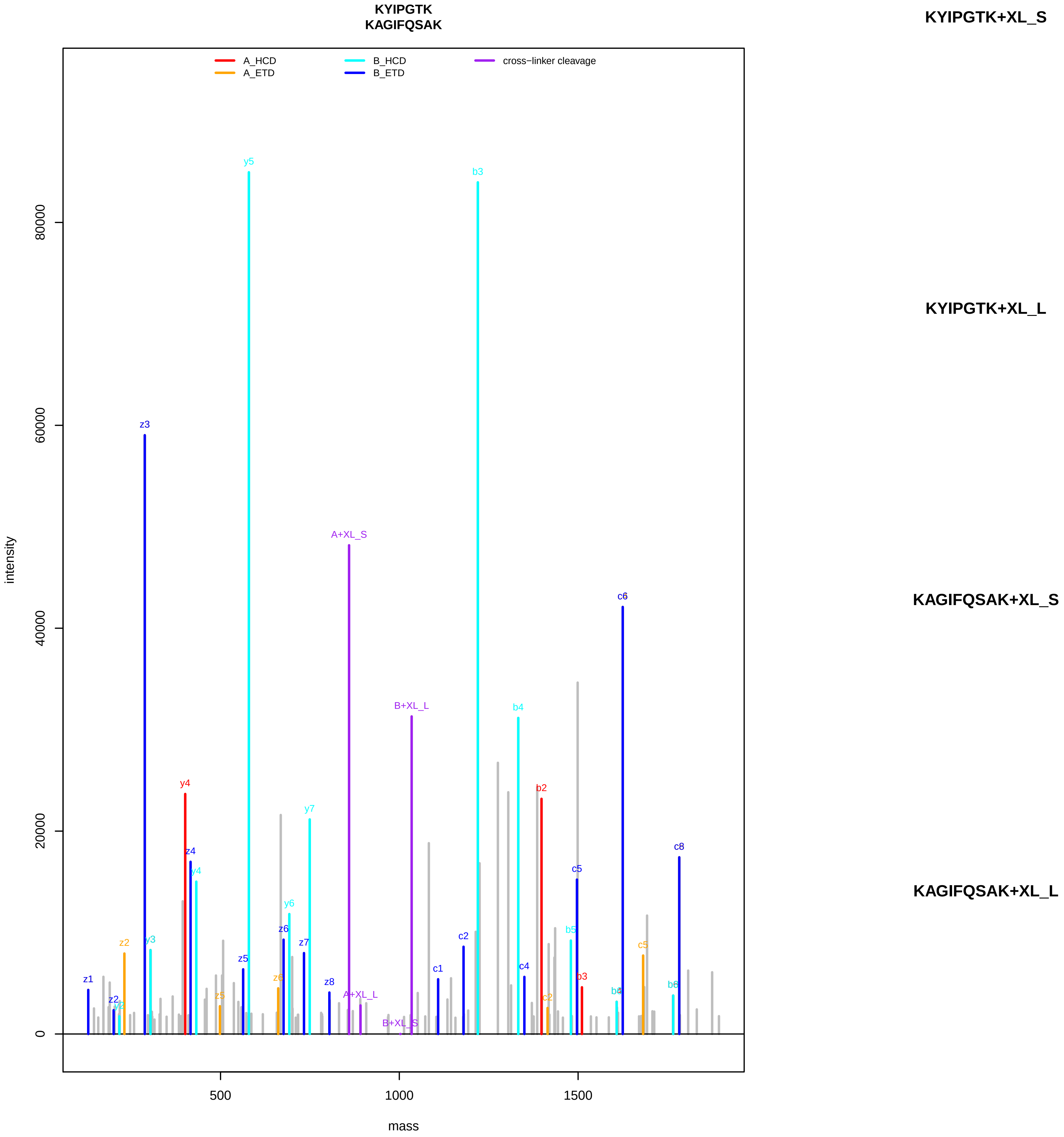
KYIPGTK
KAGIFQSAK
A_HCD
A_ETD
B_HCD
B_ETD
cross−linker cleavage
intensity
mass
0
20000
40000
60000
80000
500
1000
1500
z1
z2
y2
z2
z3
y3
y4
z4
y4
z5
z5
y5
z6
z6
y6
z7
y7
z8
A+XL_S
A+XL_L
B+XL_S
B+XL_L
c1
c2
b3
c4
b4
b2
c2
b5
c5
b3
b4
c6
c5
b6
c8
KYIPGTK+XL_S
KYIPGTK+XL_L
KAGIFQSAK+XL_S
KAGIFQSAK+XL_L
